# Refractile superficial retinal crystals and chronic retinal detachment: Case report

**DOI:** 10.1186/1471-2415-6-3

**Published:** 2006-01-13

**Authors:** Maged S Habib, Sinead Byrne, John H McCarthy, David HW Steel

**Affiliations:** 1 Sunderland Eye Infirmary, Queen Alexandra Road, Sunderland. SR2 9HP. UK; 2 Pathology Department, Gloucestershire Royal Hospital NHS Trust, Great Western Road. Gloucestershire. GL1 3NN UK

## Abstract

**Background:**

Few previous reports have described the presence of retinal refractile opacities at the macular area in patients presenting with longstanding peripheral retinal detachment. The exact nature of these opacities is unknown.

**Case presentation:**

Two patients were referred with an abnormal appearance of refractile opacities in the macular area noted during routine examination. Both were found to have longstanding peripheral retinal detachments. Subretinal fluid analysis of one patient revealed the presence of multiple birefringent crystals. We hypothesise that these crystals are the origin of the refractile macular opacities noted.

**Conclusion:**

This report describes the rare presentation of asymptomatic peripheral retinal detachment by the detection of refractile macular opacities on routine examination. It highlights the importance of meticulous peripheral retinal examination in these cases. The article also describes the findings of the subretinal fluid analysis and discusses the possible hypothesis behind their appearance.

## Background

The presence of refractile opacities located at the level of the inner retina in patients with chronic long standing peripheral retinal detachment has been described. [1-3] The exact nature of these opacities is not known. We describe two patients who presented asymptomatically with refractile opacities in the macular area in one eye on routine examination. Subsequent examination showed a longstanding peripheral retinal detachment in both cases. Microscopic examination of sub retinal fluid drained during subsequent retinal detachment repair in one of the patients showed the presence of numerous crystals, possibly calcium oxalate, suggesting that the opacities observed at the macular area were actually crystals derived from the subretinal space.

## Case report

### Case 1

**Figure 1:**
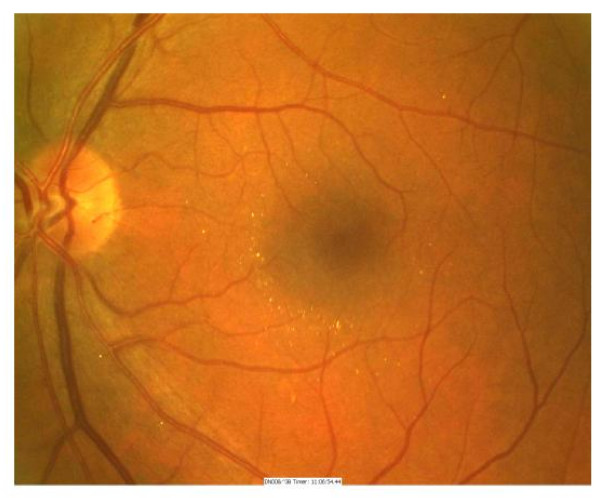
Fundus photograph of the left eye demonstrating multiple refractile glistening opacities in the peri foveal area.

**Figure 2:**
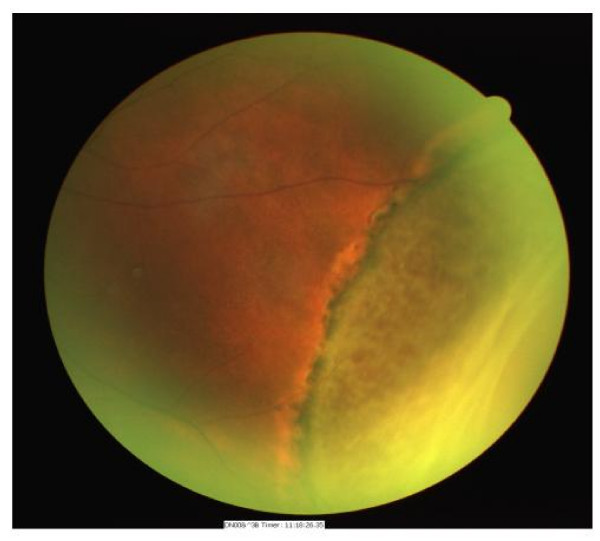
Fundus photograph of the same eye demonstrating peripheral retinal detachment secondary to a retinal dialysis.

**Figure 3:**
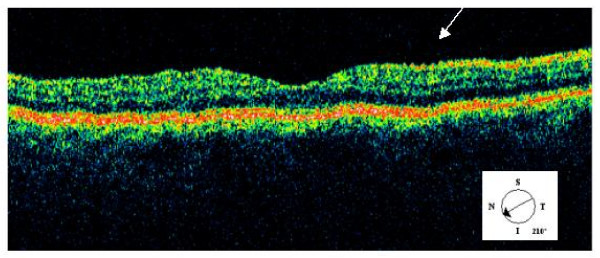
OCT image of macula showing superficial location of opacities in inner retina (arrow).

A 45-year-old man with a history of non-insulin dependant diabetes for two years attended for digital fundal photography for diabetic retinopathy screening. He was asymptomatic with 6/6 unaided vision bilaterally. Examination of the photographs on the right showed multiple refractile glistening opacities at the macula, with no apparent cause. (Illustrated in Fig. 1) The left eye was normal. The patient was referred for further eye examination. Examination at this time confirmed the finding of refractile opacities in the inner retina throughout the posterior pole but concentrated in the perifoveal area. Examination of the peripheral retina showed a longstanding retinal detachment with a pigmented tidemark, secondary to a retinal dialysis measuring two clock hours in extent. (Illustrated in Fig. 2). There were pigment cells in the anterior vitreous with occasional microscopic glistening vitreous opacities. There was no evidence of posterior vitreous separation. There was no history of ocular trauma. Optical coherence tomography confirmed the location of the opacities on the inner retinal surface. (Illustrated in Fig. 3)

Surgery to repair the retinal detachment was discussed with the patient, but further intervention was declined. Follow up one year later showed no change in the appearance of the opacities or the retinal detachment with a stable acuity at 6/6.

### Case 2

A 25-year-old man was referred by his optician, who had noted an abnormal left macular appearance during a routine check-up. He was asymptomatic and achieved unaided vision of 6/6 bilaterally. On examination, the left macula showed scattered superficial refractile deposits at the retinal surface. There was also an infero-temporal 2 clock hour retinal dialysis and secondary longstanding retinal detachment, with evidence of recent extension. The patient gave a history of being assaulted 6 months earlier. The right retina was normal. He underwent uncomplicated retinal detachment surgery with subretinal fluid drainage using a monitored drainage technique, with a 25-gauge needle externally through the sclera as previously described.[4], cryotherapy and scleral buckling. The retina was attached postoperatively and he maintained 6/6 vision during a follow-up period of 2 years.

**Figure 4:**
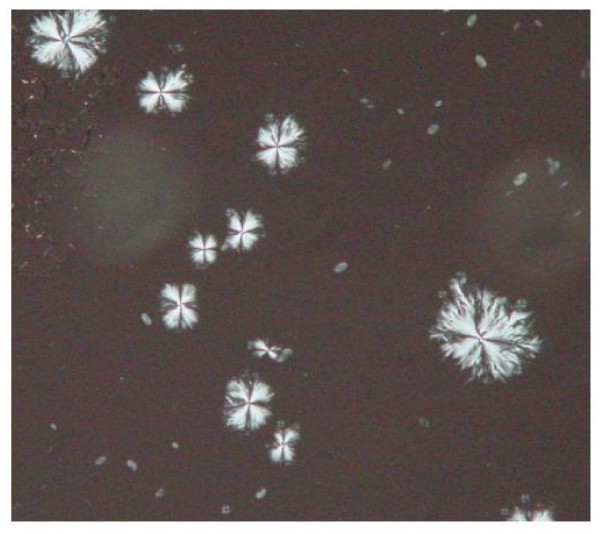
Photomicrograph of subretinal fluid showing crystals arranged in regular polyhedral shaped rosettes.

Analysis of sub retinal fluid showed multiple small brilliantly birefringent crystals under polarised light. They were unlike the large rectangular or rhomboidal shaped crystals with notched borders typical of cholesterol.[5] The crystals consisted of spindles clustered together in regular polyhedral shaped rosettes with a radiate pattern (Illustrated in Fig 4) measuring approximately 1–3 microns in size.

Both patients were completely fit and well with normal renal and liver function, good nutrition and no previous surgery or general anaesthesia. There was no history of canthaxanthin or nitrofurantoin use. Blood cholesterol and oxalate levels were normal. There was no family history of retinal disease.

## Discussion

The occurrence of inner retinal refractile deposits at the macula in patients with peripheral chronic retinal detachment has been described previously [1-3]. The unusual finding in these cases-described here was that the patients actually presented with the macular appearance and the retinal detachment was subsequently diagnosed.

The exact nature of the opacities observed in similar cases and previous case histories has been debated. [1-3] Possibilities previously proposed have been that the opacities represent haemosiderin laden macrophages, erythrocyte breakdown products or were possibly secondary to vitreous separation from the internal limiting membrane. Banaee and Ahmadieh [6] suggested that the liberation of photoreceptor outer segments particles into the vitreous cavity and anterior chamber with longstanding retinal detachments may be a mechanism and possible explanation for the refractile opacities observed by Ahmed et al.[1] and Ciardella et al.[3] The refractile opacities we have seen bear a striking resemblance to the retinal crystals observed in oxalosis,[2] and the appearance of the crystals seen on subretinal fluid analysis was consistent with that of calcium oxalate as well [5,7,8]. Unfortunately precise biochemical identification of the crystals in both cases was not possible because of the small sample sizes obtained.

Calcium oxalate has been observed in enucleation specimens of eyes with longstanding retinal detachment. [7,8] In these cases the crystals have been detected and apparently arising from the outer retinal surface of the detached retina making them invisible ophthalmoscopically. The source of oxalate in these cases has been hypothesised to occur through ascorbic acid metabolism.[7,9] Ocular fluids have an unusually high content of ascorbic acid including subretinal fluid.[10] Calcium oxalate is highly insoluble in an alkaline environment and it may be that the ph elevation associated with tissue degeneration in retinal detachment facilitates crystal deposition.[7] Furthermore the retinal pigment epithelium degeneration and subsequent liberation of melanin may be important in oxalate production in longstanding retinal detachment, especially those associated with trauma, as it has been shown that the melanin precursors phenylalanine and tyrosine can be converted to oxalate.[11] Interestingly subretinal fluid from three other patients with chronic rhegmatogenous retinal detachment but without refractile macular deposits that we have examined did not show any such crystals.

## Conclusion

These two cases describe an unusual and rare presentation of asymptomatic peripheral retinal detachment. It illustrates the importance of a thorough peripheral retinal examination to exclude retinal detachment in patients presenting with crystalline opacities at the macula.

Subretinal fluid analysis in one of the cases has allowed us to hypothesise that the crystalline opacities observed at the macula may be the same as the crystals seen in the subretinal fluid. It is possible that the crystals have migrated through the dialyses and then gravitated intravitreally, in the absence of a posterior vitreous detachment, to deposit on the vitreo-retinal interface in the macular area. The exact nature of the crystals in uncertain but it is possible that they represent calcium oxalate.

## Competing interests

The authors declare that they have no proprietary or financial interest in any aspect of this article.

This study received no public financial support

The author(s) declare that they have no competing interests.

## Authors' contributions

DHS reviewed the patients and operated upon one patient, MH and SB participated in the study design and helped to draft the manuscript. JHM performed the histopathological analysis of the subretinal fluid.

## Pre-publication history

The pre-publication history for this paper can be accessed here:


